# The impacts of the UK Global Challenges Research Fund on early career researchers: a case study

**DOI:** 10.1017/S0031182026102674

**Published:** 2026-08

**Authors:** Devon J. Chandler, Alexander I.F. Simpson, Paul W. Denny

**Affiliations:** 1 Department of Biosciences and Department of Chemistry, Durham University, Durham, UK; 2 Department of Anthropology, Durham University, Durham, UK

**Keywords:** Chagas disease, early career researchers, international research collaboration, leishmaniasis, Neglected Tropical Disease, research capacity strengthening

## Abstract

International collaborations enhance research outcomes by providing partners with increased access to resources, training and multidisciplinary expertise. These partnerships facilitate knowledge exchange and infrastructure development, promoting sustainable research ecosystems. The development of early career researchers (ECRs) is particularly important in strengthening research capacity within international research networks. However, literature regarding their experiences is limited. Here, we present a qualitative study of one international research network, the UK Global Challenges Research Fund-supported Global Network for Neglected Tropical Diseases (2018–2022) and its impact on ECR career development. Semi-structured interviews were conducted with 19 ECRs previously supported by the Network; these were then thematically analysed. Key themes were identified relating to ECR experiences with the Network. Findings indicate that the Network provided valuable opportunities for professional development, knowledge exchange and collaborative research, which participants described as transformative for their careers. However, ECRs stressed that structural barriers such as insufficient funding and job insecurity limited the Network’s longer-term impact, severely affecting ECR retention and development. These results highlight the benefits of international research collaborations and the need for targeted support and sustainable research funding to retain and develop ECRs, particularly in disease-endemic countries.

## Graphical abstract

**Figure:**
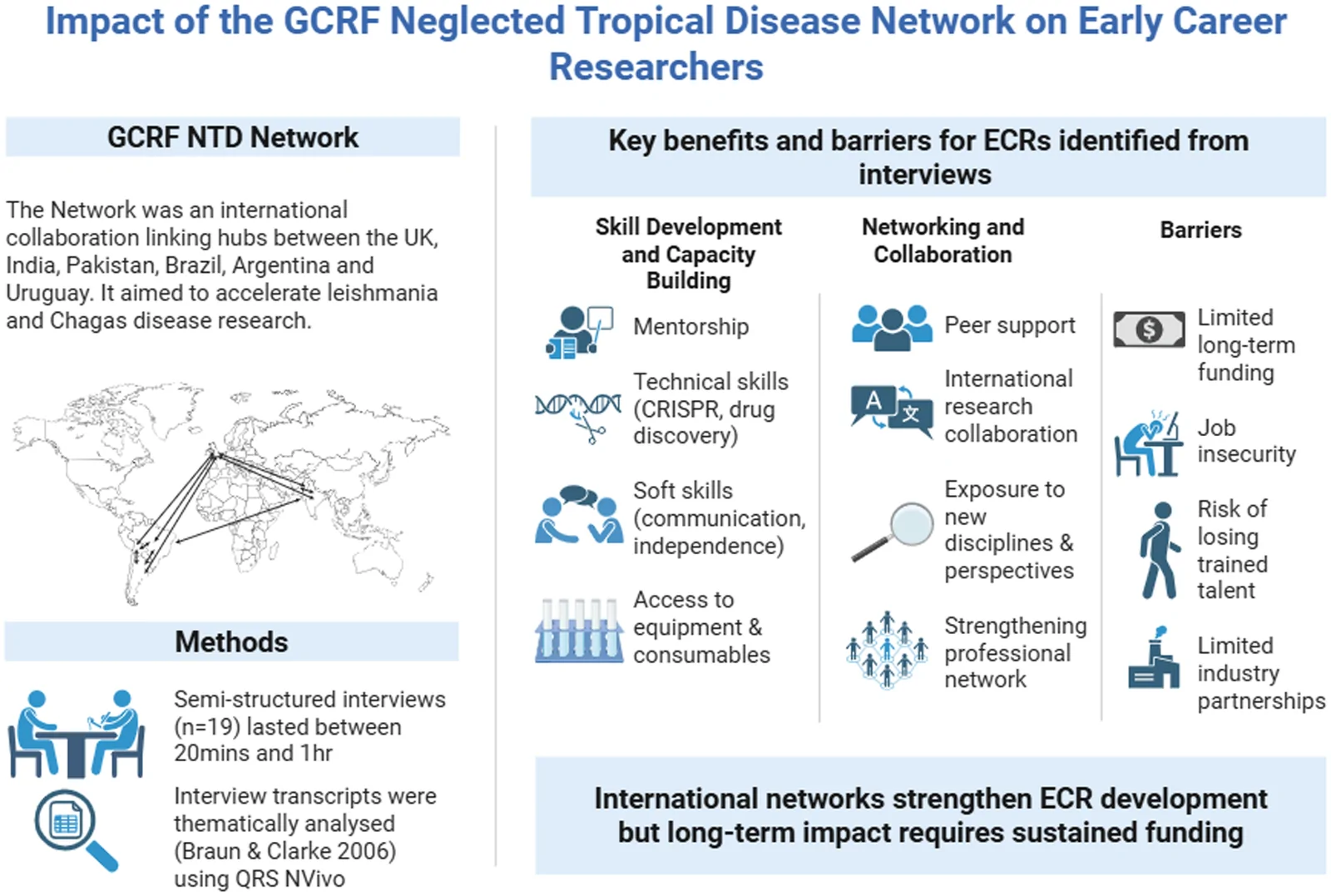

## Introduction

Establishing a research career is challenging for early career researchers (ECRs). This is particularly true in Neglected Tropical Disease (NTD) research, which, focused on diseases that impact impoverished communities in tropical and subtropical regions, is plagued by insufficient funding and poor research infrastructure in endemic areas (Hotez et al., 2009). ECR retention is essential as these researchers represent both the immediate workforce and the next generation of scientific leaders. However, limited access to training, mentorship and resources threatens ECR progression, weakening research capacity.

International research collaborations have emerged as an effective approach to address both the funding and structural challenges facing NTD research, as well as the professional development needs of ECRs (John et al., 2016). Research networks achieve this by pooling resources and facilitating knowledge exchange, for example between NTD endemic and non-endemic partners, thereby creating opportunities for ECR development and partially mitigating the impact of funding deficits. Increasingly, the success of these international partnerships is recognized as depending not only on scientific outputs but also on equitable collaboration, ensuring that research is both sustainable and relevant to endemic needs.

**Figure 1.**
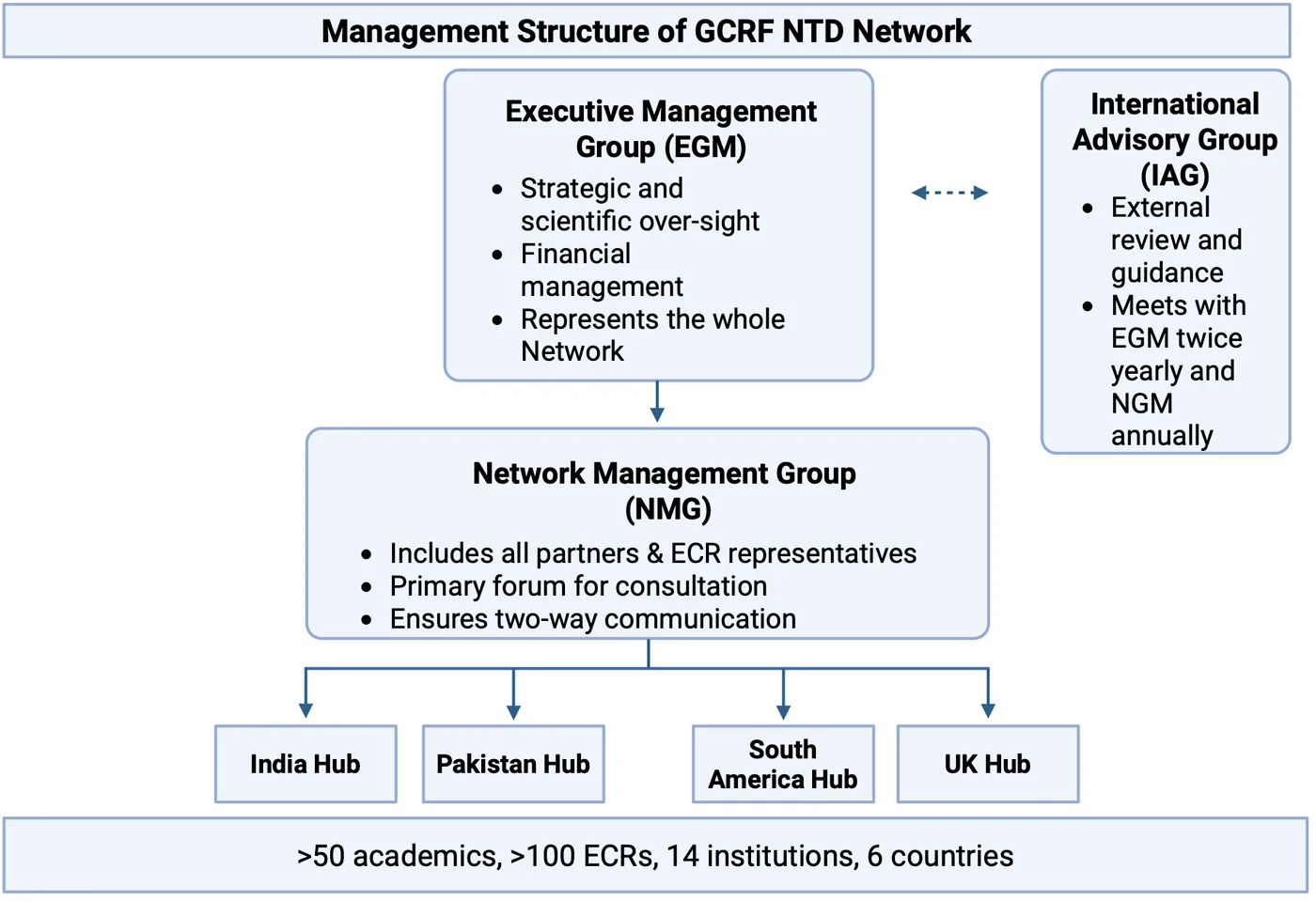
The organizational structure of the Global Network for Neglected Tropical Diseases (NTD Network). The Executive Management Group provided strategic and scientific oversight, supported by the International Advisory Group. The Network Management Group served as the main forum for consultation and 2-way communication across all partners, including ECRs. Four regional hubs (India, Pakistan, South America and the UK) led local research activities, training and capacity strengthening, feeding grassroots ideas and needs into the wider NTD Network.

The Global Network for Neglected Tropical Diseases (the NTD Network) was an international research consortium (Figure 1) that brought together over 50 academics across 14 institutions. The Network researched diagnostic and treatment solutions for 2 NTDs, leishmaniasis and Chagas disease, and supported >100 ECRs through fellowships, secondments, workshops and conference opportunities alongside their home institutions (UKRI, 2020). Leishmaniasis and Chagas disease are among the most impactful NTDs, with inadequate and often toxic treatment options (Scariot et al., 2022).

The NTD Network was funded by the UK Global Challenges Research Fund (GCRF). However, this funding stream has since been cut, and the NTD Network has ended (UKCDR, 2023).

Here, we present a case study assessing the impact of the NTD Network on the progression and retention of the ECRs involved, providing valuable insights for other research teams and funders.

## Materials and methods

This study used a qualitative research approach to explore the impact of the NTD Network on ECRs. This approach was chosen to capture in-depth, nuanced insights into the experiences, perceptions and career trajectories of ECRs supported by the NTD Network. Semi-structured interviews included open-ended questions aligned with the research aims to encourage detailed responses. Ethical approval was granted by Durham University. Participants were sent a participant information sheet before providing written consent. Participation was completely voluntary, and all data were anonymized and securely stored.

### Participant selection

Thirty-four ECRs involved in the Network were purposively selected to represent all countries involved in the Network and were contacted by email. From this initial contact, 19 interviews were arranged based on participant availability. All participants were ECRs and had a range of involvement with the NTD Network, including independent research fellows, postdoctoral researchers and PhD students. The sample was representative of all countries involved in the Network: the UK, India, Pakistan, Brazil, Argentina and Uruguay.

### Interview data

Semi-structured interviews, lasting between 20 min and an hour, were conducted in July and August 2025. All interviews were carried out via Microsoft Teams in English. Participants were asked to reflect on their experience with the NTD Network and the impact it had on them and their careers. The interview guide focused on the following 4 areas: the impact of the Network on ECR careers, the role of training and mentorship within the Network, the effects of collaboration and networking and the impact of COVID-19 on ECR experiences within the Network. The interview guide was considered for modification as the study progressed to explore additional themes raised by participants during interviews. We, therefore, added questions on the funding environment and its impact on ECR career trajectories to the interview guide, as this emerged as a key theme.

### Interview analyses

All interviews were audio-recorded, transcribed, verified with participants by the interviewer and analysed in QRS NVivo (Lumivero, 2025). All transcripts were fully anonymized. The transcripts were thematically analysed using scientifically descriptive thematic analysis. This mode of analysis understands themes as containers within which to summarize and report potentially divergent experiences. It follows the 6-step process described by Braun and Clarke (2006), with key themes and patterns generated through familiarization, coding, theme generation, review and definition, and report write-up. All coding was done by the primary researcher (D.J.C.), who had no affiliation with the Network or the participants. D.J.C. had no previous contact with any of the participants. All data were then summarized based on the themes that emerged.

## Results

**Figure 2.**
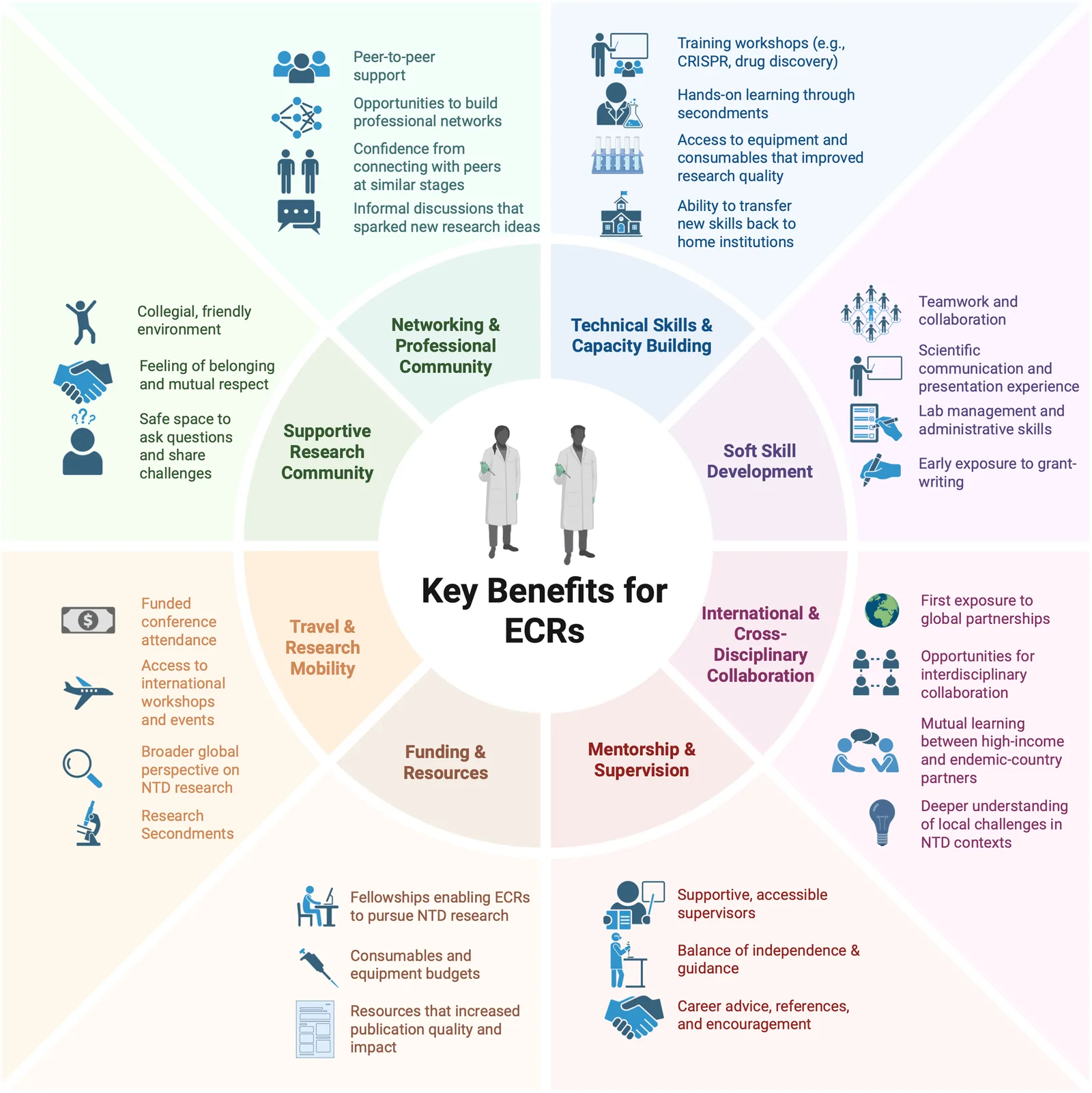
Conceptual framework derived from thematic analysis, illustrating the key dimensions of early career researcher experiences within the Global Network for Neglected Tropical Diseases. Figure 2 long description.

Thematic analysis identified 3 major themes that reflect the experiences of ECRs within the NTD Network: skill development and capacity building; collaboration and networking; and legacy and barriers to ECR retention. These themes offered insight into both the strengths of the NTD Network and the challenges limiting its impact. The key benefits identified through thematic analysis are synthesized in Figure 2, which provides a conceptual framework for the findings presented below, which are also summarized in Tables 1–3.

**Table 1.** Key subthemes within the skill development and capacity building findings Table 1 long description.

Subtheme	Description	Examples from the Network
Technical skill training and secondments	Development of laboratory and methodological skills through structured workshops, spending time in partner laboratories	CRISPR workshops, drug discovery training, parasitology skills, practical sessions in host labs, access to high-end equipment
Transferable (soft) skills development	Growth in professional competencies beyond technical ability	Communication, teamwork, lab management, scientific writing, administration
Access to equipment and resources	Improved ability to conduct high-quality research due to funding for consumables and equipment	Purchasing laboratory supplies, strengthening local lab infrastructure
Grant-writing skills	Introduction to funding structures and proposal development	Small internal grants, co-writing proposals with supervisors, managing small budgets
Increased research autonomy	Ability to manage projects independently and make informed scientific decisions	Leading experiments, planning research timelines, presenting data with confidence
Exposure to interdisciplinary approaches	Learning to work across biology, chemistry, and clinical science to tackle NTD challenges	Projects involving researchers across disciplines
Knowledge exchange and peer learning	Informal learning through discussion with peers from other institutions and countries	Peer mentoring, troubleshooting, discussion groups during workshops and conferences

**Table 2.** Key subthemes within the collaboration and networking findings Table 2 long description.

Subtheme	Description	Key examples from the Network
Peer-to-peer support	ECRs benefited from connecting with colleagues at similar stages, sharing experiences and advice	Informal discussions at conferences, WhatsApp groups, peer help with interviews and presentations
International collaboration	Opportunities to work with partners across countries, often for the first time	Joint projects between Network countries, co-supervised work, shared access to datasets and materials
Interdisciplinary interaction	Exposure to diverse scientific disciplines increased innovation and broadened understanding	Collaboration between chemists, biologists, clinicians, new research directions sparked during workshops
Confidence building through community	Supportive network environment reduced intimidation and increased ECR confidence	Recognizing ‘friendly faces’ at events, encouragement from senior researchers, feeling part of a community
Informal idea generation	Unstructured conversations led to new insights, collaborations and problem-solving	Discussions during travel, accommodation, coffee breaks, spontaneous brainstorming producing project ideas
Understanding diverse research contexts	Collaborations enhanced awareness of local challenges and realities in endemic regions	UK researchers learning about field constraints, endemic-country researchers feeling valued and recognised
Long-term professional relationships	Relationships formed in the Network often extended beyond the project’s funding period	Continued collaborations, follow-on grants, joint publications
Industry links	Some opportunities to engage with non-academic partners	Business engagement vouchers, isolated connections with industry in specific hubs

**Table 3.** Key subthemes within the legacy and barriers to ECR retention findings Table 3 long description.

Subtheme	Description	Key examples from the Network
Positive scientific and cultural legacy	The Network fostered collaborative research habits, strengthened capacity in endemic regions and demonstrated the value of equitable partnerships	Increased cross-country collaborations, improved research culture, continued professional relationships, skills embedded in home institutions
Loss of momentum without follow-on funding	Gains made during the Network risk diminishing due to lack of sustained investment	Inability to continue NTD projects, discontinued collaborations, discontinued travel and training due to funding ending
Scarcity of NTD research funding	Limited funding restricts opportunities for ECRs to stay in the field and forces them to shift research areas	‘Going where the money is’, inability to secure fellowships, leaving NTD research despite interest and expertise
Job insecurity in academia	High competition and limited stable positions discourage long-term engagement and retention	Strong candidates struggling to find permanent posts, ECRs leaving academia entirely
Weak local infrastructure	Institutions lacked equipment or resources needed to sustain advanced techniques learned during the Network	Inability to purchase consumables, reliance on Network equipment and funding, difficulty maintaining high-quality research
Challenges in industry engagement	Limited R&D capacity in certain regions and low commercial incentive for NTDs restrict translational opportunities	Lack of local industry partners, difficulty securing industrial funding for NTD-related work
Pandemic disruption	COVID-19 removed key opportunities for travel, collaboration and skills development	Cancelled workshops, delayed projects, return of unused travel funds, reduced access to lab facilities
Emotional strain	Stress related to funding insecurity, publication pressure and uncertainty about career prospects	Anxiety about producing publishable results, demotivation leading to attrition

### Skill development and capacity building

#### Mentorship and supervision

Within skill development, mentorship and supervision emerged as a key sub-theme facilitating ECR growth. All participants spoke positively about their relationships with their PrincipaI Investigator (PI)s, although their supervision arrangements varied from frequent, structured meetings to more informal contact.

ECRs valued the technical advice and problem-solving support provided by their PIs but equally emphasized the independence they were given. Multiple participants described this as *empowering*, and that ‘the level of autonomy and ownership that we had to pursue the idea that we wanted was 100%’ (P02).

Many participants felt that PIs were approachable, ‘any problem, any doubts, any advisement that I needed, [the PI] was always there to give it to me’ (P15). This helped to balance independence with support.

Participants also valued career advice provided by their PI, and many described how the references they provided were invaluable in reaching interview stages for both job and grant applications.

Interactions with PIs from other institutions were described as highly beneficial, with PIs providing further support and advice. One ECR explained that these interactions were ‘really helpful to have, as I felt that was a bit more limited in the institute I was at’ (P12). Another participant, whose PhD was co-supervised by PIs from 2 different institutions, described how ‘knowing other supervisors around the world was really good for me to have those connections’ (P01).

ECRs from endemic Low and Middle Income Countries (LMICs) highlighted the value of interactions with UK-based PIs, which gave them more confidence in their approaches and the importance of their work. P13 explained that ‘when they appreciate our work, it’s a kind of … it’s a kind of encouragement and bolsters [our] confidence’.

However, some participants noted that the frequency of these interactions and ‘this kind of interest decayed a little bit’ (P02) as the project went on. Participants felt that these interactions were incredibly valuable, and when asked what the NTD Network could have done differently, multiple ECRs suggested that ‘the major thing that we need is [to be] more [in] touch’ (P04).

ECRs consistently framed mentorship as a central part of their experience with the NTD Network, and as ‘a result of all of that mentorship, I gain at least three concrete capabilities … technical autonomy, project management, and grant readiness’ (P19).

#### Technical training and workshops

ECRs discussed the training and other development opportunities that were made available to them. NTD Network opportunities included workshops and research secondments to other institutions.

All ECRs viewed workshops as valuable opportunities. Workshops were ‘a great experience and a great exposure’ (P17) for those unfamiliar with the techniques taught. Furthermore, workshops reinforced and developed existing knowledge and skills, which had ‘a very significant effect on our understanding’ (P17) that allowed ECRs to take experimental approaches they were already familiar with and apply them to their research in different ways.

Research secondments were also described as particularly useful, giving participants access to different lab equipment and expertise. Participants described how these skills were then transferred back to home institutions, expanding local research capacity.

Not all participants felt that the training opportunities met their needs. This was in part due to the wide range of disciplines represented within the Network. One ECR explained, ‘there was a couple [of workshops] that were either very chemistry-based or … more molecular biology-based, and it just didn’t coincide well with what I was doing’ (P12).

Some participants also felt that opportunities were not shared equally. One noted that, due to an administrative error, they were left off an emailing list and, therefore, did not find out about opportunities. These cases were a source of frustration.

#### Transferable skills

In addition to technical (soft) skills, ECRs discussed that they felt the transferable skills they developed were equally important to technical skills.

ECRs valued the development of teamwork skills, and many felt that collaborative research produced more outputs of a higher quality. Being part of a network increased the scope of collaborations that ECRs were involved in, from those within home institutions to those with other institutions in the same country and internationally. Many ECRs had not experienced international collaborations, and they were keen to develop the skills it requires.

Participants saw communication skills as essential, not only to increase the quality of publications, but also to communicate findings at conferences. One ECR from an endemic country described how exposure to an English-speaking audience at one of the NTD Network conferences helped to build their confidence ‘to get rid of that initial [fear] to ask [questions in English]’ (P09). This confidence then helped them secure a position in the USA.

Laboratory management and administrative skills were areas in which many participants had no previous experience, yet many ECRs welcomed the opportunity to develop these skills, as they were seen as part of the process of establishing themselves as independent researchers. The NTD Network Fellowships came with the opportunity to apply to different defined pots of consortium funding to advance their independent research agendas. One Fellow described their experience of applying to these smaller grants, explaining it helped ‘with the process of thinking … how do you write a project, how do you get an idea that is feasible to execute? How much will it cost?’ (P11). They noted that ‘now feel I can manage my own lab when the time comes’ (P11).

#### Funding and resources

The provision of funding and resources was vital to capacity building. Indeed, without funding from the Network, many ECRs would not have had the opportunity to work on NTDs. This was especially true in resource-poor endemic settings such as Argentina, where grant funding was extremely sparse.

Participants appreciated the ‘very flexible travel money’, with one participant noting that ‘the day the GCRF [NTD Network] stopped, my international travel stopped’ (P13). Some participants had to return travel money due to the impact of the COVID-19 pandemic and travel restrictions. Participants expressed their frustrations that, due to lockdown restrictions, some of these events did not go ahead.

Participants also outlined the impact that the funding had on the quality of their research. Some institutions were able to buy new equipment, such as PCR machines, and the funding allowed them to buy more expensive consumables. One participant noted that their colleagues were able to access higher impact journals ‘because the fellowship [gave] them the consumable money part’ (P13), which has been beneficial for both the visibility of their research and their careers.

### Collaboration and networking

#### Importance of networking

Participants described networking as a central part of their experience with the NTD Network. Connecting with peers at a similar career stage allowed ECRs to share experiences and support each other. Such peer-to-peer interactions were ‘very enriching, you don’t feel so behind … When you’re talking always to researchers above you. It’s like I will never get to that point’ (P11).

In-person events, such as conferences and workshops, provided extended opportunities for informal discussions that deepened these connections. For some, these more informal interactions sparked fresh ideas that led to new research directions.

‘That networking stuff, I think that just gave me different flavour of research ideas, and then I was able to use the same tool which I knew before … to answer different questions in a more innovative way’ (P09).

Through these exchanges, particularly at conferences, participants were able to develop a wider appreciation of NTD research that had a ‘major impact in my career … to increase my view of what is going on in neglected tropical diseases in different parts of the world’ (P07). Participants explained how exposure to new perspectives helped them develop and solve problems in their own research, where ‘you can’t do it how you would have done it yourself, and so you speak to people and try different things. That’s really where the Network came in handy’ (P12).

ECRs who were able to attend multiple conferences and workshops felt that such regular interactions gave them ‘more chances to build collaborations because you’re in contact and discussing ideas all the time’ (P07).

These experiences were not universally shared, with some participants noting that they did not ‘think we had the opportunity to actively engage with establishing relationships and collaborations with bigger labs’ (P14). Another felt that while there was ‘a lot of interaction between the PIs, but there definitely there was not too much between the students or postdocs’ (P15). These participants explained that they would have really valued these interactions had they had the opportunity to experience them.

#### International collaboration

The Network facilitated international collaborations. For some, this was their ‘first exposure to work with all these international groups’ (P13). There was a general view that ‘collaborative research is much more productive than working by yourself in your lab’ (P07). Indeed, one ECR described the ‘idea of this networking for different countries [as] genius’ (P15).

Participants felt that the ‘Network was a very good place to explore new avenues of synergy because, at the end, we were receiving funding from the same source’ (P02). Participants described how collaborations benefited all sides as by ‘putting together people from areas where the funding and the resources are less accessible than in the UK, for example, but they have other resources like access to the clinicians, access to the patient samples’ (P02). These collaborations were described as ‘game chang[ing]’ (P04), opening doors to new contacts and opportunities.

‘I have contact with people in Brazil, people in Argentina that we can share papers with, discuss ideas. Also, they exchange with GSK or with the team in Durham and York … I never had this kind of opportunity before, so that was for me a big game changer’ (P04).

International collaborations broadened ECRs’ understandings and perspectives regarding their research area. ECRs ‘had the opportunity to discuss the limitations of each country and handling the diseases and what research need to be done to increase the chances of have better medicine or better diagnostics’ (P07). It was felt that understanding local realities was fundamental to developing research that was transferable and would have an impact on those suffering from these diseases.

Many collaborations that started with the NTD Network have continued, and participants ‘still benefit [from] that connection’ (P06). ECRs explained that, now they have seen the impact of such collaborations, they ‘want to continue to do that, get involved in networks and contact people from different parts of the world and have this collaborative research’ (P07).

ECRs based in the UK saw Durham as the leading partner in the Network (Figure 1). However, participants based in endemic countries did not feel that Durham was the sole leading partner and praised the hub system as ‘work[ing] really well because they had regional leaders’ (P07). Having these regional leads was important as ‘some demands and some necessities … [you] only notice if you are in a place … it’s hard [for] people [to] say what we need and it’s hard for us here to say what people in India need’ (P07).

When asked what could have been done differently in these collaborations, ECRs wished that other institutions in more isolated regions could be involved. It was felt that these institutions would benefit greatly from such collaborations and that this would help develop endemic research capacity.

#### Industrial partners

The NTD Network also facilitated some opportunities for industry collaboration. A few participants were able to use business engagement vouchers to develop small projects with industry partners.

Although the importance of working with industry partners was widely recognized, ECRs explained that this was dependent on local R&D capabilities. One participant noted that, while they would have liked to develop a collaboration with industry, ‘they don’t have a strong industry of research and development here in Brazil’ (P07).

#### Supportive

Alongside formal collaborations, participants also described the supportive and collegial atmosphere created by the NTD Network. Having ‘friendly faces’ at conferences was reassuring (P01), and peers were described as approachable and generous with their time.

Participants felt that this created an incredibly supportive culture within the NTD Network. ECRs particularly valued this, as many described embarking on an academic career as slightly daunting, explaining that ‘it’s good to be able to ask people how it works and what to expect’ (P12).

### Legacy and barriers to ECR retention

#### Funding landscape and job insecurity

Despite the opportunities the NTD Network created, participants emphasized that the broader funding environment remained a critical barrier to a career in NTDs. Several described the absence of long-term funding and grant availability as the main reasons they ‘can’t continue working with [NTDs] for now … just because we don’t have funding’ (P04) since the Network ended. Others also felt that ‘as an academic, you have to go where the money is and the opportunity at the moment is just not really there [for NTDs]’ (P15).

Participants felt the scarcity of funding made working ‘way more stressful’. I can remember standing by pieces of equipment or opening certain data files and having absolute dread. Like, is this going to give a good result? … Is it going to help me get this paper out?’ (P05). Without these results and papers, ECRs felt they would not be able to secure further funding. Indeed, one participant noted that this working environment played a role in their decision to leave academia altogether.

Participants also noted the challenges engaging with industrial collaborators. One explained that securing money for NTD research was a ‘hard sell’ as ‘it’s difficult for [an industrial collaborator] to leverage any money that isn’t going to go to a project that’s going to directly affect their current portfolio of drugs … [which] are normally really expensive things for Western diseases’ (P18). Another described the challenges securing funding for NTD research more starkly, simply stating, ‘nobody cares’ (P15).

Participants universally felt ‘there is no such thing as job security in universities’ (P14). They felt this was true regardless of candidate quality and ‘brilliant people, very hard workers with an excellent CV are struggling to find any stable job or career’ (P01). This environment severely impacted ECR retention as ‘without the stability of the funding and jobs, it’s difficult for people to commit to just doing this research’ (P15).

#### Impact of the COVID-19 pandemic on the Network

The Network ran from 2017 to 2022, and the COVID-19 pandemic had a major impact on ECRs’ experiences. Participants reported significant restrictions on access to laboratory facilities, although these varied by country, explaining that they were ‘limited in terms of working in wet labs’ (P19). ECRs instead focused their efforts on tasks that could be conducted remotely, such as manuscript preparation, data analyses and the consolidation of existing findings.

The impact of the pandemic was not uniform across participants. Individual experiences varied, depending on research stage and project timelines. For some, particularly those who had already gathered a lot of experimental data, the pandemic provided an opportunity to focus on writing and analyses. As one participant reflected, ‘for me it was very positive … I had just finished a big bulk of *in vivo* work’ (P12). In contrast, others faced setbacks due to reduced laboratory access.

The pandemic also impacted ECR networking and communication. With in-person interactions no longer possible, ECRs relied on virtual platforms. Online meetings became central for research presentations and project coordination. However, it was universally felt that these were less effective than in-person interactions in producing new ideas and insights.

Beyond its immediate effects on research practices, the pandemic had significant implications for the sustainability of the Network itself. Participants linked COVID-19 to broader funding constraints, which ultimately contributed to the premature termination of the GCRF funding stream.

#### Legacy

Participants reflected on the legacy of the NTD Network with both optimism and concern. On one hand, the Network was seen as ‘a nice picture of the possibility that we have … to gain knowledge, tools and capacity to solve problems’ (P04) by bringing together researchers from different countries and disciplines. Similarly, another participant emphasized the Network’s success in fostering collaboration between ‘developed and underdevelopment countries … and how you can benefit from [this] interaction’ (P04).

However, participants stressed that ‘without follow on funding … the legacy is lessened’ (P05). Another reflected ‘the Network doesn’t really impact the funding that is available to work on the diseases, so in that sense, I don’t think the Network … had an impact’ (P12).

Multiple participants explained that their research directions had since shifted away from NTDs. It was universally agreed that funding, not technical know-how or interest, was the largest barrier to ECR retention and NTD research.

## Discussion

A central part of the NTD Network’s aims was to strengthen and progress NTD research by providing ECRs with opportunities to work on Chagas disease and leishmaniasis. These opportunities included mentorship, funded research positions, workshops, conference attendance, research secondments and travel bursaries. The findings discussed here demonstrate the transformative potential of international research collaborations to develop ECRs.

Mentorship in the NTD Network arose organically through supervisory relationships, with the formality of the relationship varying between ECRs and institutions. ECRs consistently described mentorship as a central part of their experience, and they valued both the technical guidance and the autonomy their PIs gave them. The dual emphasis on independence and support is consistent with established frameworks of research ownership in academic development (Patsali et al., 2024) and reflects findings from the Global Bridges programme (Uwizeye et al., 2020), namely that ECRs placed greater value on individual mentoring compared to workshops, social events, and study visits.

The convergence across different ECR programmes suggests that one-to-one mentorship carries particular developmental weight. Given the importance of mentorship to ECR development, future international collaborations of this kind should consider embedding mentorship arrangements into the programme design rather than leaving them to emerge naturally.

Technical training through workshops and secondments was another capacity-building mechanism of the NTD Network, and participants broadly valued these. Skills transferred back to home institutions represent what Krauss et al. (2023) describe as a multiplier effect, expanding capacity at the institutional rather than purely individual level. The disciplinary breadth of the Network meant that not all individual ECRs felt the available workshops were relevant to their needs, analogous to findings in other interdisciplinary programmes (Uwizeye et al., 2020). However, interdisciplinary exposure was also one of the greatest benefits for ECRs, leading to different perspectives, new research ideas and better problem-solving skills.

One gap that participants identified in the NTD Network was the insufficient development of non-technical skills, particularly grant-writing. Some participants felt this would have been particularly helpful, and these skills are essential for research success (Acharya et al., 2023; Bonaconsa et al., 2024). The NTD Network focus on technical skills, while valuable, missed an opportunity to fully prepare ECRs for the competitive funding environment they would face after the programme ended. Indeed, guidelines developed by representatives from 12 ECR networks (Kaikkonen et al., 2025) recommend that ECR development frameworks include training in these competencies.

The Network was designed to foster international research collaboration, and the findings indicate that it achieved this. ECRs described international collaborations as transformative, particularly those who had not worked internationally before. Many collaborations that started through the Network persisted after its end, a positive indicator of the Network’s impact. The current study did not include a bibliometric analysis of NTD Network ECR outputs to quantify this, representing a limitation that future evaluations of such programmes should address.

Peer-to-peer networking among ECRs emerged as a particularly valued dimension of the NTD Network experience, providing both practical support and reassurance. This reflected previous studies (Suarez et al., 2023) that identified connections with peers at similar career stages as a distinct and irreplaceable form of support that is particularly beneficial for ECRs during periods of uncertainty. The informal interactions at in-person events such as conferences and workshops were consistently described by NTD Network ECRs as catalysts for new ideas and directions.

The Network’s hub-and-regional-leads structure was praised by participants in endemic countries, and this is worth recognizing as a project design success. Historically, North–South research partnerships have been criticized for extractive dynamics in which LMIC partners provide access to clinical material and patient populations while High Income Country partners drive intellectual and methodological agendas (John et al., 2016). The NTD Network’s regional structure partially countered this by embedding local leadership into the Network’s architecture, strengthening both the representation of endemic-country researchers in decision-making and their agency to influence the programme’s implementation. These findings suggest that equitable partnerships depend not only on technical capacity building but also on governance arrangements that represent all partners, enabling local needs to shape the direction and delivery of international research programmes.

The COVID-19 pandemic also played a large role in ECR experiences of the Network. Those participants who had already completed experimental work found the period productive for writing and analyses, while others faced significant delays due to restricted laboratory access. The transition to online interactions was seen as a practical but inferior substitute for in-person collaboration, particularly for the informal exchanges that generated new ideas and created and sustained collegial relationships.

Through the Network, participants developed technical skills, built international networks and developed confidence as independent researchers. By these measures, the Network was a success. However, despite the Network’s success in developing individual ECR competencies, the difficulty securing follow-on funding has forced many ECRs to leave NTD research despite expertise and interest, reflecting the well-documented global neglect of NTD research (Hotez et al., 2009; Wolfe et al., 2024). Although short programme cycles of 3–5 years are sufficient to train ECRs in technical skills and build collaborations, they do not create the job environment that will retain those ECRs. The result is a cycle of ECR development and capacity building followed by talent attrition.

The findings of this study add to a growing body of evidence that systemic reform of research funding models is necessary to retain ECRs in research (Daniels, 2015; de Winde et al., 2021; OECD, 2021). Without reform, even well-designed programmes will continue to produce capable ECRs who change career pathways due to the job insecurity they face in their field. Furthermore, ECRs in the Network suggested it could have included more isolated institutions and explained that the impact on those institutions would have been transformational. These institutions were not included in the Network, as limited infrastructure and research capacity would have constrained their ability to contribute meaningfully within the programme’s budget. However, if capacity building is a core objective of such networks, grants must be large enough so that even isolated institutions can be included.

The NTD Network achieved many of its capacity building objectives: ECRs developed skills, established lasting collaborations and gained confidence as independent researchers. However, the Network’s sustainability is threatened by the wider funding environment. Many ECRs have left NTD research, not by choice but because funding opportunities are unavailable. This is a waste of talented researchers and programme investment. Without sustained investment, momentum will be lost.

### Study limitations

It should be noted that the study was conducted in 2025, 3 years after funding for the NTD Network had concluded. Many participants had completed their work with the Network prior to this, and as a result several struggled to recall precise details about their experiences. While the interviewer (D.J.C.) had no prior contact with any of the participants, he was associated with the NTD Network’s Director (P.W.D.). It should be acknowledged that this may have influenced participants’ responses or perceptions.

## Conclusion

To conclude, this study set out to explore the experiences of ECRs within the GCRF NTD Network. The study demonstrated both the role that international research networks can play in developing ECRs, and in advancing and accelerating NTD research. By providing mentorship, training, collaboration and access to resources, the NTD Network empowered ECRs to take ownership of their research and develop their independence. At the same time, the findings underscore the impact of the funding landscape and the insecurity of academic career pathways on NTD research. Without systemic change, there is a real risk that these challenges will erode the gains achieved by the NTD Network, leading to the attrition of talent from a field already constrained by chronic underinvestment.

## Data Availability

The datasets generated and analysed during the current study are not publicly available due to maintaining the confidentiality of individual participants. Anonymized data may be available on request.

## Figure 2 Long description

The infographic presents key benefits for Early Career Researchers (ECRs) divided into six categories: Networking & Professional Community, Technical Skills & Capacity Building, Soft Skill Development, International & Cross-Disciplinary Collaboration, Travel & Research Mobility and Funding & Resources. Networking & Professional Community includes peer-to-peer support, opportunities to build professional networks and informal discussions that spark new research ideas. It emphasizes a collegial, friendly environment and the feeling of belonging and mutual respect. Technical Skills & Capacity Building highlights training workshops (e.g., CRISPR, drug discovery), hands-on learning through secondments and access to equipment and consumables that improve research quality and impact. It also mentions the ability to transfer skills back to home institutions. Soft Skill Development focuses on teamwork and collaboration, communication and presentation experience, lab management and administrative skills and early exposure to grant-writing. International & Cross-Disciplinary Collaboration offers first exposure to global partnerships, opportunities for interdisciplinary collaboration and learning from high-income country researchers. It also includes a better understanding of NTD challenges. Travel & Research Mobility covers funded conference attendance, access to international workshops and research visits and secondments. Funding & Resources provides fellowships enabling ECRs to pursue NTD research, consumables and equipment budgets and resources that increase research quality and impact. Mentorship & Supervision includes supportive, accessible supervisors, balance of independence and guidance, career advice, references and encouragement.

Navigate back to Figure 2.

## Table 1 Long description

Three columns list Subtheme, Description, and Examples from the Network for skill development and capacity building. Row 1, Technical Skill Training and Secondments, describes laboratory and methodological skills gained through workshops and time in partner laboratories, with examples including CRISPR workshops, drug discovery training, parasitology skills, practical sessions in host labs, and access to high end equipment. Row 2, Transferable (Soft) Skills Development, covers professional competencies beyond technical ability, with examples communication, teamwork, lab management, scientific writing, and administration. Row 3, Access to Equipment and Resources, describes improved ability to conduct high quality research due to funding for consumables and equipment, with examples purchasing laboratory supplies and strengthening local lab infrastructure. Row 4, Grant Writing Skills, describes introduction to funding structures and proposal development, with examples small internal grants, co writing proposals with supervisors, and managing small budgets. Row 5, Increased Research Autonomy, describes managing projects independently and making informed scientific decisions, with examples leading experiments, planning research timelines, and presenting data with confidence. Row 6, Exposure to Interdisciplinary Approaches, describes working across biology, chemistry, and clinical science to tackle NTD challenges, with examples projects involving researchers across disciplines. Row 7, Knowledge Exchange and Peer Learning, describes informal learning through discussion with peers from other institutions and countries, with examples peer mentoring, troubleshooting, and discussion groups during workshops and conferences.

Navigate back to Table 1.

## Table 2 Long description

Three columns list each Subtheme, its Description, and Key Examples from the Network. Peer-to-Peer Support is described as ECRs connecting with colleagues at similar stages, with examples including informal discussions at conferences, WhatsApp groups, and peer help with interviews and presentations. International Collaboration covers first time opportunities to work with partners across countries, including joint projects between Network countries, co supervised work, and shared access to datasets and materials. Interdisciplinary Interaction describes exposure to diverse scientific disciplines, with examples of collaboration between chemists, biologists, and clinicians, and new research directions sparked during workshops. Confidence Building Through Community describes a supportive environment reducing intimidation, with examples of recognising friendly faces at events, encouragement from senior researchers, and feeling part of a community. Informal Idea Generation describes unstructured conversations leading to insights and collaborations, with examples during travel, accommodation, and coffee breaks, and spontaneous brainstorming producing project ideas. Understanding Diverse Research Contexts describes awareness of local challenges in endemic regions, with examples of UK researchers learning about field constraints and endemic country researchers feeling valued and recognised. Long-Term Professional Relationships describes relationships extending beyond the project funding period, with examples of continued collaborations, follow on grants, and joint publications. Industry Links describes some engagement with non academic partners, with examples of business engagement vouchers and isolated connections with industry in specific hubs.

Navigate back to Table 2.

## Table 3 Long description

Columns are Subtheme, Description, and Key Examples from the Network. Positive Scientific and Cultural Legacy is described as fostering collaborative research habits, strengthening capacity in endemic regions, and demonstrating equitable partnerships, with examples including increased cross country collaborations, improved research culture, continued professional relationships, and skills embedded in home institutions. Loss of Momentum Without Follow On Funding notes that gains risk diminishing without sustained investment, with examples including inability to continue NTD projects, discontinued collaborations, and discontinued travel and training due to funding ending. Scarcity of NTD Research Funding describes limited funding restricting opportunities for ECRs to stay in the field, with examples including, Going where the money is, inability to secure fellowships, and leaving NTD research despite interest and expertise. Job Insecurity in Academia describes high competition and limited stable positions, with examples including strong candidates struggling to find permanent posts and ECRs leaving academia entirely. Weak Local Infrastructure describes lack of equipment or resources to sustain advanced techniques, with examples including inability to purchase consumables, reliance on Network equipment and funding, and difficulty maintaining high quality research. Challenges in Industry Engagement describes limited regional research and development capacity and low commercial incentive for NTDs, with examples including lack of local industry partners and difficulty securing industrial funding for NTD related work. Pandemic Disruption describes COVID 19 removing opportunities for travel, collaboration, and skills development, with examples including cancelled workshops, delayed projects, return of unused travel funds, and reduced access to lab facilities. Emotional Strain describes stress related to funding insecurity, publication pressure, and career uncertainty, with examples including anxiety about producing publishable results and demotivation leading to attrition.

Navigate back to Table 3.
